# A Multilevel Perspective on the Health Effect of Social Capital: Evidence for the Relative Importance of Individual Social Capital over Neighborhood Social Capital

**DOI:** 10.3390/ijerph18041526

**Published:** 2021-02-05

**Authors:** Susan Lagaert, Thom Snaphaan, Veerle Vyncke, Wim Hardyns, Lieven J. R. Pauwels, Sara Willems

**Affiliations:** 1 Department of Public Health and Primary Care, Faculty of Medicine and Health Sciences, Ghent University, 9000 Ghent, Belgium; Veerle.Vyncke@UGent.be (V.V.); Sara.Willems@UGent.be (S.W.); 2 Department of Criminology, Criminal Law and Social Law, Faculty of Law and Criminology, Ghent University, 9000 Ghent, Belgium; Thom.Snaphaan@UGent.be (T.S.); Wim.Hardyns@UGent.be (W.H.); Lieven.Pauwels@UGent.be (L.J.R.P.); 3 Master of Safety Sciences, University of Antwerp, 2000 Antwerp, Belgium

**Keywords:** health status disparities, social capital, social determinants, neighborhoods

## Abstract

Employing a multilevel perspective on the health effects of social capital, this study analyzes how individual and neighborhood differences in self-rated health in Ghent (Belgium), relate to individual and collective social mechanisms, when taking demographic and socioeconomic characteristics of individuals into account. This study estimates the health effects of social trust, informal social control and disorder at the neighborhood level and social support and network size at the individual level, using indicators indebted to both the normative and resource-based approaches to social capital. Instead of the mere aggregation of individual indicators of social capital, this study uses the key informant technique as a methodologically superior measurement of neighborhood social capital, which combined with a multilevel analysis strategy, allows to disentangle the health effects of individual and neighborhood social capital. The analysis highlights the health benefits of individual social capital, i.e., individual social support and network size. The study indicates that controlling for individual demographic and socioeconomic characteristics reduces the effect of the neighborhood-level counterparts and the neighborhood characteristics social trust and neighborhood disorder have significant, but small health effects. In its effects on self-rated health, social capital operates on the individual level, rather than the neighborhood level.

## 1. Introduction

In the last decades, there has been a burgeoning academic interest in neighborhood effects on health on health [1,2]. The increasing scientific attention in public health research to how the neighborhood context affects a person’s health status is necessary as research underlines a clear spatial clustering of problems of health and wellbeing in certain neighborhoods within cities across the world [3,4,5,6,7]. Neighborhood characteristics, such as deprivation, are shown to negatively correlate with various health outcomes, including life expectancy [8,9], mental health problems [3] and self-rated health [10,11]. 

In an effort to explain health inequalities between neighborhoods, scholars have looked at the clustering of health-related resources. A large body of research has turned to socioeconomic resources as potential mechanisms to explain health differences between neighborhoods, and socioeconomic deprivation of neighborhoods and inhabitants is consistently related to worse health [8,10,11,12,13]. Additionally, researchers have looked at social resources and how they might explain health differences between neighborhoods [2,3,14,15,16,17]. 

Researchers often draw on the concept of ‘social capital’ to address how unequal social resources may explain neighborhood differences in health [2,15]. The definition of ‘social capital’ tends to vary, but it is generally related to various aspects of a person’s embeddedness in social relations and context, such as frequency of social interactions, social support, generalized trust and social cohesion [18]. Different research traditions indicate that social capital operates both on the individual and the neighborhood level [2,15,19,20]. Social capital at the individual level is thought to affect a person’s health status through access to individual social resources, such as individual social support [21,22]). As such, social capital can function as an attribute of an individual [2,19].

Social capital at the neighborhood level is also relevant for public health research [16,17,23,24,25]. Rather than an individual characteristic, neighborhood social capital is an attribute of the neighborhood that may affect a person’s health status above and beyond the individual social resources a person has access to [26,27]. Neighborhood social capital is a form of collective social capital at the level of local neighborhoods and communities; it is considered accessible through residence in a neighborhood-even though there are potential benefits for people from outside of the neighborhood, such as the safety effects of social control-and it is related to “*norms of reciprocity, civic participation, trust in others, and the benefits of membership*” ([28], p. 661). Literature reviews suggest that perceived social capital at the neighborhood level has some positive effect, albeit fairly little and not consistently, on various individual objective and subjective health indicators [15,29]. However, especially for self-rated health often a positive impact of neighborhood social capital is uncovered. Moreover, neighborhood social capital is found to be associated with (mental) health and mortality [3,16,25,30,31,32,33], as well as with detrimental health-related behaviors such as alcohol consumption and smoking [9,24,34,35,36,37].

Consideration of the relevant operational level of social capital is important because neighborhood differences can reflect both differential composition of the neighborhood (e.g., a concentration of resource-poor individuals or vice versa a clustering of inhabitants with beneficial individual social capital in a certain neighborhood) or ‘true’ contextual effects of social capital-related mechanisms operating at the neighborhood level [38,39]. Therefore, information on social capital at the individual level is necessary to fully understand the relationship between health and social capital at the collective level and vice versa ([40], p. 34).

Given the interrelation of social capital at different operational levels, scholars increasingly emphasize the importance of studying social capital in general and neighborhood social capital in particular within a multilevel framework [40,41,42,43]. This means that when evaluating the health effects of social capital across neighborhoods one cannot limit attention to either individual social resources or to neighborhood social connections, but both individual and neighborhood-level social processes should be taken into account at the same time [2,41,42,43]. In this regard, the often-used practice to measure neighborhood social capital as the mere aggregate of individual scores conflicts with the multilevel nature of social capital and its health effects and, instead, independent measurement methods for neighborhood social capital are methodologically superior [14,44,45]. 

The aim of this study is, therefore, to evaluate *the effect of individual and neighborhood social capital on individual self-rated health from a multilevel perspective*. In particular, drawing on Bourdieusian and Putnamian social capital theory, collective efficacy theory and broken windows theory, this study focuses on the health effects of various neighborhood-level and individual-level indicators of social capital. Using the key informant technique to measure neighborhood social capital and employing indicators that correspond to different conceptualizations of social capital, this study contributes to the understanding of health inequalities across neighborhoods in public health research, by presenting an application of a multilevel perspective on social capital [2].

### 1.1. Different Approaches to and Conceptualizations of Social Capital

When trying to understand the effects of social capital on the self-rated health of inhabitants in different neighborhoods, it is important to acknowledge the different traditions in social capital theory, that have different approaches to and conceptualizations of social capital. First of all, the level on which social capital operates and, thus, should be measured and analyzed, is contested [18,19,43,46]. One tradition considers social capital as an *individual* resource and one tradition interprets the concept as an asset of communities at the *collective* level. 

American political scientist Robert Putnam formulated a highly influential definition of social capital at the collective level [40,47]. By defining social capital as “*features of social organization such as networks, norms, and social trust that facilitate coordination and cooperation for mutual benefit*” ([48], p. 67), Putnam considers social capital to be a collective asset which is inherently positive [40,49]. Rather than an individual resource which is embedded in a person’s social network, research in a Putnamian tradition highlights the benefits of social connections at the community level. 

Literature that views social capital as an individual construct [50,51] is in general strongly influenced by the work of French sociologist Pierre Bourdieu ([40], p. 31). Bourdieu defines social capital as the “*sum of the resources, actual or virtual, …. by virtue of possessing a durable network of more or less institutionalized relationships of mutual acquaintance and recognition*” ([52], p. 119). Even though Bourdieu deems social capital an individual resource, it cannot be “owned” by individuals as would be possible for monetary capital. Precisely the connections to others are at the core of social capital; individual social capital vanishes when social relationships dissolve [40,53].

In the discussions on the level on which social capital operates, one notices the traces of another, conceptual, divide in the literature. Many empirical studies on social capital, including studies on social capital and health, focus heavily on social norms within networks, such as trust and reciprocity, as the core of social capital [43,47]. This *‘normative’ perspective* mostly draws on Putnam’s work and highlights the benefits of social capital. However, this focus has been subject to critique since it easily ignores the potential disadvantages of social capital [23,49] (for a review of the negative effects of social capital, see the work of Villalonga-Olives and Kawachi [54]). Indeed, it can also negatively affect health and well-being [46,54,55], for instance when there is pressure on group members to conform to undesirable norms [56]. As a consequence, some researchers have proposed a switch in social capital theory from a ‘normative’ to a ‘resource-based’ definition of social capital [2,51,53]. The latter conceptualization of social capital is indebted to Bourdieu’s legacy. 

### 1.2. Health Effects of Neighborhood and Individual Social Capital: The Studied Social Processes

- Neighborhoods & social capital: the studied social processes

The Putnamian tradition dominates the literature on the public health effects of neighborhood social capital [57]. According to the Putnamian approach, cohesive processes, such as social trust, are at the core of the neighborhood’s social capital and can foster health benefits [2,58]. Carpiano on the contrary, has made an innovative contribution by distinguishing ‘social cohesion’ and ‘social capital’ [2]. Carpiano follows and scales up Bourdieu’s resource-based approach to social capital when he argues that the cohesive mechanisms, such as social trust, Putnam identifies as social capital are actually a prerequisite for social capital, rather than capital itself. Social cohesion is a necessary condition for neighborhoods to build resource-generating assets, such as social leverage and informal social control, which can benefit health [2]. 

This reasoning aligns with the collective efficacy theory. Collective efficacy refers to “*social cohesion among neighbors combined with their willingness to intervene on behalf of the common good*” ([59], p. 918). The two primary dimensions of collective efficacy are (1) *social trust* (which Carpiano would define as ‘social cohesion’ [2]), and *informal social control* (which Carpiano highlights as an element of social capital [2]). Neighborhood social trust is a prerequisite for informal social control, and as a consequence for neighbors’ “*willingness to intervene on behalf of the common good*” ([59], p. 918, [45]). 

Theoretically, positive health effects of social trust and informal social control can be expected, as scholars hypothesize that neighborhoods high in collective efficacy lead to living contexts that are better able to acquire resources in domains of health, housing and education, with fewer risks and more safety nets [60] (p. 186). A recent review article finds evidence for a positive effect of neighborhood social trust on self-rated health [29]. The effect of informal social control on self-rated health is less frequently analyzed and the concept is often used in composite measures. The self-rated health effects of neighborhood informal social control appear more inconsistent, as some researchers find significant positive effects on self-reported mental health [61] and on self-rated health, albeit for specific segments of the population [60], and others do not find a significant effect on self-rated health when controlling for confounders [23,62] or a negative effect [63]. 

In this study both social trust and informal social control are focused on, as is neighborhood disorder. Neighborhood disorder is associated with different aspects of the social organization of neighborhoods ([37], pp. 53–54), such as neighborhood social ties and social support [64] and social cohesion and feelings of unsafety [65]. Generally, higher levels of neighborhood disorder are related to lower levels of social cohesion and social control on deviant behavior [27,37,66]. Neighborhood disorder is central to contemporary social disorganisation theory [27,67], applied evolutionary ecology [68], the ‘broken windows’ theory by Wilson and Kelling [69] and Skogan’s ‘disorder and decline’ model ([70], p. 219). Neighborhood disorder has both physical aspects, i.e., the level of physical stress a neighborhood suffers as a consequence of, for instance, exhaust fumes, noise, littering and odor nuisance [37,71], and social aspects. Signs of social disorder, that are examined in this study, include people selling drugs on the street in a neighborhood, or people harassing passers-by, or fighting on the street and other tangible signs of disorder; thus, disorder affects feelings of unsafety [65] and the extent to which people rather avoid a certain neighborhood [45]. Neighborhood disorder is shown to be detrimental for a person’s (physical and mental) health [16,64,65,72,73]. Ross and Mirowsky report that “*the daily stress associated with living in a neighborhood where danger, trouble, crime and incivility are common apparently damages health*” ([73], p. 258). The negative (mental) health effects of disorder appear not only to be direct but also indirect, as the negative correlation is mediated by perceived cohesion ([72], pp. 302, 309) and neighborhood social ties and support ([64], p. 268). Bjornstrom and colleagues indicate that cohesion only protects against poor health when inhabitants live in neighborhoods with low to moderate disorder; when there is high neighborhood disorder, cohesion loses its beneficial effect on health [72].

- Social capital at the individual level: the studied social processes

From a Bourdieusian perspective, *individual* social capital may influence health [72]. Bourdieu pays attention to both the quantity and quality of network connections ([74], p. 249). Not only the volume or amount of network ties a person can mobilize (“quantity”), but also the resources a person’s network can provide (“quality”) are of interest [2,71]. Therefore, this study pays attention to two aspects of individual social capital: individual social support and the individual’s social network size. Social support concerns the support and help by/from social ties within and outside of the neighborhood a person experiences. Social support is a resource for the individual as it benefits physical and mental health and well-being [75,76]. The social network size of the individual refers to the number of social contacts a person has on a daily basis [77]. According to Fu, this straightforward single-item measure correlates highly to more complex indicators to measure network size [77].

### 1.3. Conceptual Model and Hypotheses

**Figure 1:**
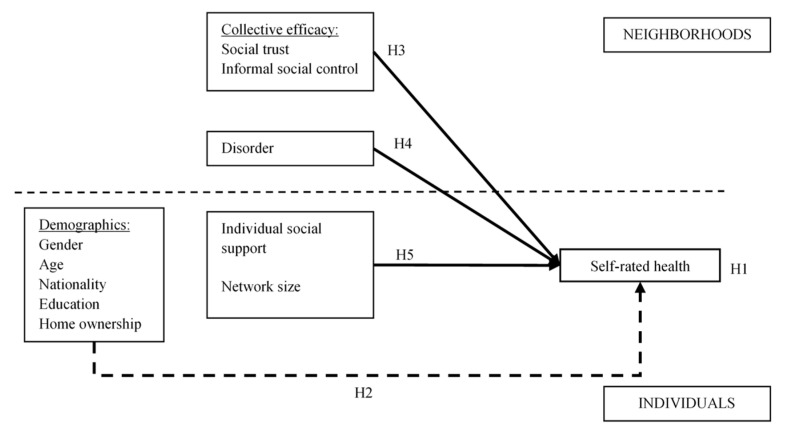
Contextual model–Hypothesized effects on self-rated health.

The hypotheses tested in this study are summarized in Figure 1, representing the contextual model. These hypotheses are derived from the theoretical framework that integrates insights from social capital theory and the different conceptualizations of social capital, collective efficacy theory, and broken windows theory and uses a multilevel perspective on social capital and its effects on health:

**Hypotheses 1** **(H1).** Significant variation in self-rated health can be attributed to the neighborhood level.

**Hypotheses 2** **(H2).** The variation in self-rated health at the neighborhood level exists independently of the composition of the neighborhood (i.e., socio-demographic and socioeconomic characteristics of individuals).

**Hypotheses 3** **(H3).** Higher neighborhood social trust and higher informal social control are associated with higher levels of self-rated health, independent of the neighborhood’s (socio-demographic and socioeconomic) composition.

**Hypotheses 4** **(H4).** Higher neighborhood disorder is associated with lower levels of self-rated health, independent of the neighborhood’s (socio-demographic and socioeconomic) composition (a), and neighborhood disorder reduces the effects of neighborhood social trust and informal social control (b).

**Hypotheses 5** **(H5).** An individual’s social support (a) and an individual’s network size (b) are positively associated with self-rated health, after taking into account the individual’s socio-demographic and socioeconomic background and neighborhood processes.

## 2. Materials and Methods 

The data collection for this study took place in Ghent. Ghent is the second-largest city of Belgium. The city is located in the northwest of Belgium with 257,945 residents in 2016 (Stad Gent, 2017) and a surface of 156 km² (±1653 residents/km²). The data were collected as part of the Social capital and Well-being In Neighborhoods in Ghent (SWING) study, for which financial support was provided by the Research Foundation—Flanders (FWO; Grant Number GA05511N_SVDS_2922 and FWO12/ASP_H/295). The used methodology and measurements in the SWING study are already described in detail in other publications [45,78].

### 2.1. SWING Study

The SWING study consists of three successive cross-sectional waves of data collection in neighborhoods in Ghent in 2011, 2012, and 2013 ([45], p. 1001). Neighborhoods were operationalized using statistical sectors. The statistical sector level is comparable to the census tract level in the US or the UK and is the smallest administrative unit of analysis on which administrative data (demographic, social, and economic variables) are available [45,78]. Scarcely populated neighborhoods (with less than 200 adult inhabitants) were not included for methodological reasons (to avoid large measurement errors [79]) and for content-related reasons (because the aim of the study was to measure the association between individual and neighborhood *social* processes). Data are available for all neighborhoods that are inhabited by at least 200 adults, so this study contains the total population of (significantly) inhabited neighborhoods in Ghent (*N* = 142) ([45], pp. 1002–1003). 

In each cross-sectional wave, multiple data collection methods were applied. Next and for each level of the multilevel model, the methodology and measurements are reported; more information can be found elsewhere ([45], pp. 1001–1002). The *level-1 measurement (i.e., sampling of inhabitants)* employs data which were collected using a representative survey based on face-to-face interviews with neighborhood residents. Face-to-face structured questionnaires and a short self-administered questionnaire (for some possibly sensitive questions, for instance, about substance use and income) were employed for the data collection. For each neighborhood a randomized stratified sample was drawn from the municipal registry. This sample was stratified by age, sex, and current nationality. The inclusion criteria to participate as a neighborhood inhabitant in the survey were: (1) being 18 years old or older, (2) not living in an institutional setting (such as a prison or retirement home), and (3) having sufficient knowledge of Dutch, which is the language spoken in Ghent, to complete the survey. Comparisons between the reached sample and the initial randomized stratified sample suggest that there is a slight underrepresentation of individuals with a non-Belgian nationality in the reached sample. The aim was to reach 20 inhabitants in each of the 142 neighborhoods. In total, 2730 neighborhood inhabitants participated [78].

The *level-2 measurement (i.e., sampling of key informants)* employs data which were collected via the key informant technique by using standardized questionnaires for the measurement of neighborhood social processes. Key informants are “*persons who are in a ‘privileged’ position to provide detailed information on local processes*” ([44], p. 404). They are field experts due to their profession (e.g., police officer or social worker) or their -in another way acquired- expertise. The key informant technique offers an alternative for simply aggregating the individual-level responses to neighborhood mean scores, to prevent an accumulation of the measurement error at the individual level ([45], p. 1002). Hardyns and colleagues argue: “*In most studies, the community-level measures of social capital are simply the aggregates of the individual-level measures (using mean scores). However, independent measurement methods for neighborhood social capital should be preferred*.” ([78], p. 3). Additionally, Pauwels and Hardyns point out that *“… the knowledge of well-chosen key informants about the social climate of an area is superior to the knowledge of the average inhabitant of that area*” ([44], p. 402). They also demonstrate that, if a careful and diverse choice of key informants is made, key informants can fill the gap when measuring processes in local contexts. Thus, key informants can provide high-quality information that describes community and neighborhood organizational processes. In the SWING study, the researchers aimed for a heterogeneous set of 8 to 10 key informants per neighborhood. The inclusion criteria for key informants were: (1) being 18 years old or older, (2) having sufficient knowledge of Dutch, which is the language spoken in Ghent, to complete the questionnaire, and (3) being in a position that infers an above average knowledge of the social processes in one of the studied neighborhoods. In total, 1400 key informants participated [78]. 

### 2.2. Measures

The *dependent variable* is self-rated health. This concept was measured by asking the respondents to rate their general health status, using a 5-point Likert scale ranging from ‘very bad’ to ‘very good’ [40]. A higher score refers to a better health status. This subjective health indicator has consistently been associated with objective measures of mortality and morbidity and is therefore considered as a valid measure of health status [80,81,82].

The *independent variables at the individual level (level-1)* include the measurement of individual social capital and the control variables (c.q. socio-demographic and socioeconomic variables); the latter are used to differentiate contextual effects from neighborhood compositional effects [42,78]. The measurement of the different indicators is also reported in detail elsewhere: ([45], p. 1003). Two types of control variables are included to represent the composition of the neighborhood. First, the socio-demographic variables ‘age’ (as a continuous variable, in years), ‘gender’ (0 = male, 1 = female), and ‘nationality’ (0 = other nationality at birth, 1 = Belgian nationality at birth) are used. Second, the socioeconomic variables ‘education’, which relates to the respondents’ highest obtained degree, (1 = lower level of secondary education (similar to junior high school in the U.S.), 2 = higher secondary education (similar to high school in the U.S.), 3 = higher education/post-secondary education), and home ownership (1 = tenant in social rented housing, 2 = tenant in privately rented housing, 3 = owner) are included. Individual social support was measured by asking the respondents to indicate on several items how many friends, family members or acquaintances would give practical support. Examples of items are: “How many friends, family members or acquaintances would let you move into their house for a week if you temporarily could not stay at your house?” and “How many friends, family members or acquaintances would encourage you to go to the doctor if you experience health problems?” (on an 8-point scale, ranging from nobody to more than 10 people). These items were summed to create a scale with higher scores indicating higher levels of social support (see Table A1 for individual items and results of factor and reliability analysis). The individual’s network size is measured by asking inhabitants with how many people they have social contact on an average weekday. This variable is incorporated in the model as a trichotomized measure, divided into tertiles (1 = ≤10 persons, 2 = 11–25 persons, 3 = ≥26 persons).

The *independent variables for the neighborhood-level social capital measures (level-2)* were created through the afore-mentioned key informant technique to measure neighborhood social processes. Neighborhood (perceived) social trust is based on the key-informants’ assessment of items such as: “People around here are willing to help their neighbors” or “People in this neighborhood can be trusted” (on 5-point Likert scales, ranging from totally disagree (1) to totally agree (5)). Higher scores indicate more neighborhood social trust. Neighborhood informal social control is based on the key-informants’ assessment of the likelihood that neighbors would intervene when certain negative things happen in their neighborhood such as: “… when children were spray-painting graffiti on a local building” or “… when a fight breaks out in front of their house” (on 5-point Likert scales, ranging from very likely (1) to very unlikely (5)). Higher scores on the variable indicate lower neighborhood social control. Neighborhood disorder is based on the key-informants’ assessment of how often certain negative things happen in their neighborhood, such as: “Groups of adolescents harassing people to obtain money or goods” or “People being threatened on the streets with weapons or knives” (on 5-point Likert scales, ranging from never (1) to very often (1)). Higher scores on the variable point to higher neighborhood disorder.

A two-step approach was applied in constructing the neighborhood-level measures [83]. First, the key informant responses on above-mentioned individual items in the standardized questionnaires were summed to scales to measure neighborhood social trust, neighborhood informal social control and neighborhood disorder at the individual level of key informants. Second, the individual scores on these scales were summed and aggregated to the neighborhood level for the specific neighborhood about which the key informant shared his/her knowledge. This happened after checking and with respect for the ecological reliability. All level-2 indicators are scale constructs, namely social trust, informal social control, and disorder; these were also described in another publication: [45] (p. 1004). In Table A1, the individuals items, as well the results of factor analysis and reliability analysis of these level-2 scale constructs can be found. 

### 2.3. Analytic Strategy

SPSS Statistics (version 25, IBM Corporation, Armonk, NY, USA) is used to estimate a two-level hierarchical linear regression model with individuals at level 1 and neighborhoods at level 2 ([45], p. 1004). This multilevel analysis accounts for the nested data structure, which is characterized by individuals living within neighborhoods, and makes it possible to estimate (1) the effect of individual and neighborhood-level indicators on self-rated health (fixed part) and (2) the variation in self-rated health among neighborhoods that cannot be accounted for by the included independent variables (random part). Thus, multilevel analysis allows to evaluate the extent to which potential neighborhood differences in self-rated health are due to either compositional (individual-level) characteristics or contextual (neighborhood-level) characteristics. 

First, an intercept-only model (so called “null model”) is estimated, without any level-1 or level-2 predictors. Only individual-level socio-demographic and socioeconomic variables (control variables or confounders) are included in model 1 to determine to what extent differences in self-rated health between neighborhoods can be explained as a compositional effect. Model 2 includes the two key dimensions of collective efficacy [59], i.e., social trust and informal social control, which are important elements in social capital theory [2]. In model 3, disorder is added to check to what extent this variable is associated with self-rated health and to evaluate the net-association of social trust and informal social control with self-rated health, both independent of neighborhood composition. Next, the effect of individual social capital on self-rated health is evaluated. Individual social support and the individual’s network size, both measured at the individual level, are included in model 4 to verify whether these variables are associated with lower or higher levels of self-rated health, when taking into account the socio-demographic and socioeconomic background of individuals, and aspects of neighborhood social capital, namely social trust, informal social control, and disorder at the neighborhood-level. The metric variables were included as standardized variables in the analysis to allow for effect comparisons. 

The reported deviance-statistic allows to assess the improvement of model fit. This statistic was obtained by a likelihood ratio test. The deviance-statistic follows a chi-square distribution with degrees of freedom equal to the difference in the number of parameters estimated in the two models [45,84,85]. We checked for multicollinearity and noticed a very high correlation between neighborhood socioeconomic disadvantage and disorder (*r* = 0.75, *p* < 0.001). Also the correlation between neighborhood socioeconomic disadvantage and social trust was high (*r* = −0.55, *p* < 0.001). Because of multicollinearity problems, neighborhood socioeconomic disadvantage was not included in the multilevel analysis. None of the variables included in the estimated models indicated a variation inflation factor (VIF, which signals potentially problematic inflation of the standard errors due to multicollinearity problems) above the critical factor 4 (all under 1.6).

## 3. Results

The descriptive statistics of the characteristics of the 2730 inhabitant-respondents and 142 neighborhoods are presented in Table 1. The sample is equally distributed when it comes to gender: 48% of the sample were men and 52% of the sample were women. The mean age of the respondents is 48 years and ranges from 18 to 95. Almost 10% of respondents had a non-Belgian nationality at birth. The majority of the sample is home owner (almost 70%). From the inhabitants of the sample who rent, 10% lives in social rental housing and 20% lives in private rental housing. Somewhat less than half of the sample has obtained a degree in higher education. Self-rated health consists of a single-measure item on a 5-point Likert scale where a higher value refers to a higher self-rated health. The mean self-rated health and the corresponding standard error indicates that inhabitants with a high self-rated health are in the majority. Self-rated health has a significant negative skewed distribution (skewness = −0.87, standard error of skewness = 0.05).

The bivariate correlations between the neighborhood-level characteristics are shown in Table 2. Social trust and disorder are significantly correlated (r = −0.57, *p* < 0.001). On the contrary, social trust and informal social control weakly correlate (r = 0.13) and this correlation is not significant (*p* > 0.05). This demonstrates that both variables measure separate dimensions of neighborhood social capital. The two individual-level parameters of different dimensions of social capital (i.e.,: (1) individual social support and (2) the individual’s network size) show a significant, but relatively small positive correlation (Spearman’s ρ = 0.349, *p* < 0.001). This relatively small association shows that both variables measure separate dimensions of individual social capital, indicating the existence of a multidimensional concept of social capital at the individual level.

Table 3 displays the results of five successive models for self-rated health in accordance with the formulated hypotheses. Model 0 is an intercept-only model, without explanatory variables. The results in Table 3 indicate that there is a small but significant variation in self-rated health at the neighborhood level, as predicted by Hypothesis 1. The Intra-class Correlation Coefficient (ICC) is 2.86%, which means that 2.86% of the observed individual differences in self-rated health can be attributed to the neighborhood level. 

In model 1, demographic and socioeconomic control variables (confounders) are included and the variation at the neighborhood level is reinterpreted. Because adding the background variables reduces the ICC to 1.18% and because the model fit significantly improves, model 1 shows that a significant amount of the variation at the neighborhood level is due to the differing composition of neighborhoods in terms of these demographic and socioeconomic variables. This indicates that it is necessary to exclude composition effects before estimating the true contextual effects of neighborhood social trust, informal social control and disorder. As can be observed, the neighborhood random effects are no longer significant, in contradiction to what was expected (H2). The estimates show that, on the individual level, women, the elderly, people with low levels of education, and tenants report lower levels of self-rated health. The nationality of inhabitants does not significantly affect self-rated health. 

From model 2 onwards, the neighborhood (level-2) characteristics are included in the analysis. In model 2, social trust and informal social control are introduced. Social trust has a small but significant positive effect on the level of self-rated health. In other words, inhabitants of neighborhoods characterized by higher levels of social trust also report significantly higher levels of self-rated health. The effect of informal social control is not significant. So, there is partial support for Hypothesis 3. Due to the introduction of these neighborhood-level characteristics, the between-neighborhood variance in self-rated health is substantially reduced and, consequently, the ICC further shrinks to less than a half percent. Now, 0.46% of the observed individual differences in self-rated health can be explained by the characteristics at the neighborhood level. The deviance-statistic indicates that the model fit is significantly improved. 

In model 3, neighborhood disorder is included as an independent variable at the neighborhood level. The net-effect of disorder is in line with Hypothesis 4a: higher levels of disorder are associated with lower levels of self-rated health. This association is significant (*p* < 0.01). This neighborhood measure has on its own an impact on the model, since the ICC is further reduced to 0.24%; however, adding this variable does not lead to a significant improvement of the model fit. The introduction of disorder at the neighborhood level slightly decreases the impact of neighborhood social trust on self-rated health, suggesting that the effect of social trust is partially mediated by disorder (Hypothesis 4b). 

In model 4 the individual-level indicators of social capital, i.e., individual social support and the individual’s network size are added to test Hypotheses 5a and 5b. Hypothesis 5a is supported as people who report higher levels of individual social support also report higher levels of self-rated health. As can be observed, also network size on the individual level has a small, but significant effect on self-rated health (Hypothesis 5b). People with few ties report lower self-rated health than people with a large network (26 or more). However, compared to inhabitants with large network, the results do not indicate lower self-rated health for people with a moderate network size (11–25 contacts). The values of several compositional variables, such as education and home ownership, are reduced when introducing the individual-level indicators of social capital to the model, which means that, previously, these compositional variables were partially capturing the effect of individual social capital. Taking individual social support and the individual’s network size into account significantly improves the model fit, compared to the previous model. Adding both indicators to the model at the individual level results in an increase of the ICC, which means that the importance of the characteristics at the neighborhood level increases. When a comparison of the effect sizes is made (made possible because the metric variables were included as standardized variables), neighborhood social trust and neighborhood disorder have a similar effect on self-rated health (as the coefficients have absolute values of 0.044 and 0.045, respectively), but the effect of individual social support is clearly larger (as the coefficient has an absolute value of 0.108). 

To be certain that the uncovered effects are not due to the fact that linear hierarchical models were performed, an additional hierarchical ordered logit model was estimated that treats self-rated health as an ordinal variable. This analysis that uses ‘very good self-rated health’ as a reference category and includes standardized metric predictors to allow for effect comparisons, is presented in Table A2. The ordered logit analysis (in Table A2) confirms the findings of the final linear hierarchical model (model 4 in Table 3). With regard to the neighborhood-level indicators: the higher the social trust in the neighborhood people live in, the less likely these inhabitants are to report worse self-rated health compared to ‘very good self-rated health’ (*p* < 0.05); the higher the level of disorder in the neighborhood people live in, the more likely these inhabitants are to report worse self-rated health compared to ‘very good self-rated health’ (*p* < 0.05). The neighborhood level of informal social control does not predict individual self-rated health. Also the findings regarding the individual-level indicators in the ordered logit analysis (shown in Table A2) lead to similar conclusions as were drawn from model 4 of Table 3: persons are less likely to report worse self-rated health compared to ‘very good self-rated health’ when their individual social support is higher (*p* < 0.001). With regard to network size, the results indicate that respondents with a very small network are more likely to report worse self-rated health compared to ‘very good self-rated health’ (*p* < 0.01). When a comparison of the effect sizes is made, neighborhood social trust and neighborhood disorder have a similar effect on self-rated health (as the coefficients have absolute values of 0.102 and 0.110, respectively), but the effect of individual social support is clearly larger (as the coefficient has an absolute value of 0.291). 

## 4. Discussion

This study evaluates the effect of individual and neighborhood social capital on the self-rated health of adult inhabitants across neighborhoods in Ghent, Belgium. By doing so, it builds upon the call to analyze social capital from a multilevel perspective [41,42,43]. Drawing both on Bourdieusian and Putnamian social capital theories, collective efficacy theory and broken windows theory, this study estimates the health effects of social trust, informal social control and disorder at the neighborhood level and social support and network size at the individual level, using indicators indebted to both the normative and resource-based approaches to social capital. 

As expected, this study indicates that the self-rated health individuals report varies across neighborhoods. Differential neighborhood composition explains neighborhood differences in self-rated health. This means that the fact that people with similar demographic backgrounds (e.g., age, nationality, gender) and socioeconomic backgrounds (e.g., level of education and living in a socially rented, privately rented or self-owned home) cluster in certain neighborhoods explains variation in self-rated health across neighborhoods. Moreover and in line with other studies [86], this study highlights the importance of individual social capital, showing that social support positively affects self-rated health. Furthermore, people with a small social network report lower self-rated health than people with a large number of ties. This is found when taking the demographic and socioeconomic background of individuals into account.

However, although to limited extent, some elements of neighborhood social capital seem to contribute to self-rated health across neighborhoods. While neighborhood social trust benefits individuals’ self-rated health as hypothesized, a higher informal social control did–unexpectedly- not contribute to better health. As expected, higher neighborhood disorder is associated with lower self-rated health. Moreover, disorder accounts to a small extent for the positive effect of neighborhood social trust, but because the test of model improvement lacked statistical significance, one should be careful not to draw far-reaching conclusions in this respect. All in all, in its effects on self-rated health, social capital operates more on the individual level, than on the neighborhood level in this study, as is also illustrated by the comparison of effect sizes.

This study has some important strengths. Firstly, our analyses include different indicators of social capital, according to both the normative and resource-based perspectives, thereby incorporating competing conceptualizations of social capital and acknowledging the complex nature of the concept, a point made by Vyncke ([40], p. 177). The combination of both definitions and conceptions of social capital provides a more thorough understanding of the relationship between social capital and self-rated health and counters the predominating ‘normative’ approach to social capital that extensively focuses on social norms such as trust and reciprocity [43]. Secondly, consistent with the multilevel framework, this study examines both individual and neighborhood-level social capital, which is necessary as individual and neighborhood social capital can function as two distinct constructs that have diverging health effects [30,40]. Thus, this study contributes to the understanding of the multilevel nature of social capital and its effects on health, using a multilevel perspective on social capital that acknowledges both the individual and neighborhood level as a relevant operational level. Thirdly and in agreement with the multilevel perspective, neighborhood capital is measured employing the key informant technique. This approach is both statistically and methodologically superior to measurement strategies that treat neighborhood social capital as a mere aggregate of individual scores [44,78]. This adds to other strengths of the study design, including the large study size and the labor-intensive respondent selection and data collection method (interview in the home of the respondents, repeated attempts to contact respondents, …) which reduces selection bias.

In spite of its contributions, we want to add some critical notes that should be addressed in future research. A limitation of this work relates to the inability of the used cross-sectional study design to pinpoint the direction of causality between social capital and health [87,88]. Indeed, while certain components of social capital, such as a person’s network size, may affect health, also the opposite could be true, which would mean that an individual’s poor health results in smaller social networks. It requires longitudinal data, such as panel data, to solve this causality question. However, it is unsure that even longitudinal data would necessarily allow to identify the actual nature of the association between social capital and health, as they probably have a bidirectional and mutually reinforcing, or circular, relationship [89]. The slight underrepresentation of people with a non-Belgian nationality in the reached sample (compared to the drawn randomized sample) potentially makes the generalizability of the findings to the entire population, including people with a non-Belgian nationality, difficult. It is important to note, however, that the aim of this study was not to evaluate the divergent health effects for different ethnic groups, so the implications of this slight underrepresentation are rather limited. Nevertheless, it is clear that future research should try to guarantee the representativeness of the used study sample for people with other ethnicities and nationalities, by avoiding that language difficulties cause drop-out, for instance by providing questionnaires in different languages ([40], pp. 223–224). 

Furthermore, one has to acknowledge that, all in all, this study did not present large effects of neighborhood social capital on self-rated health. There are several possible explanations. First, different overlapping contexts, such as neighborhoods, work settings and school settings, influence health [90]. As a consequence, the findings could reflect a mismatch between the context(s) relevant for the study participants’ health and the used operationalization of neighborhoods, as also explained by Vyncke ([40], p. 175). Second, earlier research suggested that the absence of significant health effects of social capital at the collective level could be explained by the interaction between individual and collective social capital [19], but this potential cross-level interplay of social capital at the individual and the collective level is not considered in the present study. Third and related, it is possible that social capital has health benefits for specific subpopulations. For instance, neighborhood social capital may only have health effects for certain ethnic or SES groups or depending on the SES of the neighborhood [14,16,91]. Attention to these diverging (interaction) effects would also allow to dig deeper into the possibility that specific manifestations of neighborhood social capital, such as neighborhood informal social control, have opposite effects in particular segments of the population, that balance each other out and sum up in very small net effects in the overall population. This could potentially clarify why findings for different manifestations of social capital, such as neighborhood informal social control, are so inconsistent between studies [15,29]. As Villalonga-Olives and colleagues [92] argue, evaluating which segments of the population may disproportionally benefit from certain social capital interventions is important to enhance public health (p. 203). 

Fourth, the relatively minor effects of neighborhood social capital could be related to the subjective measure of health status used in this study. In fact, there is some evidence that the match between self-rated and actual health depends on the social circumstances of a person [93,94]. Neighborhood characteristics could be associated more with objective health status, rather than with subjective health status, as people in resource-poor neighborhoods may take their resource-poor neighbors with a bad health status as a reference point and, thus, overestimate their true health status when they are reporting their self-rated health (e.g., reference group theory [95]), or because expectations about health increase with the educational level ([93], p. 429). This would mean that neighborhood social capital has a more pronounced effect when studying specific health behaviors, such as smoking or physical activity, or objective indicators, such as mortality risk. Fifth, it is also possible that neighborhood social capital is associated more with a person’s mental health than with physical health, as suggested by research by Ziersch and colleagues [32]. 

Sixth, it is possible that research in other country-contexts, such as the USA, finds larger effects of neighborhood social capital. Indeed, one could question the relevance of focusing on the neighborhood level in research within the European context [96]. After all, the socio-spatial segregation between neighborhoods in Europe is less pronounced than in the USA [97], where the research tradition focusing on neighborhood effects originates from [96]. Last, it is possible that other neighborhood characteristics than social capital cause differences in self-rated and objective health across neighborhoods. For instance, the socio-spatial quality of the neighborhood, such as walkability or the availability of parcs, may explain health inequalities across neighborhoods [98].

Further research should further clarify these expectations regarding the effects of neighborhood social capital and other neighborhood characteristics on objective health status, health behaviors and mental health, for specific subpopulations. Future research should also address one of the major challenges for contextual neighborhood effect studies, which relates to the myriad of ways social capital is conceptualized and operationalized and the obstacles this poses to the scientific understanding of which manifestation of social capital has the most direct and objective impact on which health indicator, for which segments of the population, in which context and through which mechanisms. Indeed, even after decades of research, it is still unclear which manifestations of neighborhood social capital always protect health or protect health more than others, as literature reviews reveal differences between health indicators, point to large inconsistencies between studies, show the health effects of different elements of neighborhood social capital to be context-dependent or different for subpopulations and signal that social capital indicators are too often used in composite measures, making disentangling the effects of different nuances of neighborhood social capital impossible [15,57,99,100]. A clearer understanding of the exact health effect for the different social capital indicators used in the literature is necessary to substantiate the implications of social capital research and research on neighborhood effects for public health improvements. Therefore, we reiterate the call of other scholars that more attention should go to differentiating between divergent conceptualizations of social capital in order to find common ground and make comparison possible [29,57]. Future research could also elaborate on the multilevel perspective on social capital applied in this study by focusing on other indicators of neighborhood social capital, such as community social support, that were not addressed in this study [2]. As such, this study and future research on this topic add to the understanding of how strengthening social capital of individuals in neighborhoods can lead to better health outcomes. Indeed, this study highlights that reinforcing the social support and the social network of individuals is an important element to improve the health of inhabitants across neighborhoods. As a consequence, health improvement programs should pay attention to individual social capital when addressing health inequalities across neighborhoods. Moreover, this study indicates that to improve health promoting social capital as a health-related resource is probably not enough. Interventions that focus on social capital to advance health are “*a complement to, rather than a replacement for, broader structural interventions*” ([101], p. 185). Socioeconomic health-related resources such as educational attainment, which Mirowsky and Ross [102] and Link and Phelan [103,104] argue is the fundamental cause of health, and which proves to be an important predictor of health in the analyses of this study as well, are crucial when enhancing good health across neighborhoods.

## 5. Conclusions

This study evaluates the effect of individual and neighborhood social capital on self-rated health. In particular, drawing on Bourdieusian and Putnamian social capital theory, collective efficacy theory and broken windows theory, this study focuses on the health effects of neighborhood social trust, informal social control and disorder at the neighborhood level and social support and network size at the individual level. Thus, this study contributes to the growing literature on the health effects of neighborhood characteristics, by presenting an application of a multilevel perspective on social capital [2]. The study specifically highlights the importance of individual social capital, as the estimated neighborhood-level social processes are either non-significant (as is the case for informal social control), or have a smaller effect on self-rated health (as is the case for neighborhood social trust and neighborhood informal social control). This study calls for further research using a multilevel perspective on the effects of social capital on health behaviors and objective health indicators and for different segments of the population. More work is also needed on one of the great challenges for neighborhood social capital research, which refers to the myriad of ways social capital is conceptualized and operationalized and the obstacles this poses to the scientific understanding of which manifestation of social capital has the most direct and objective impact on which health indicator, for which segments of the population, in which context and through which mechanisms. Every study on health effects of neighborhood social capital, also this study, is confronted with this hiatus in the literature and, more importantly, this issue jeopardizes the application of findings for the improvement of public health. Therefore, we reiterate the call of other scholars that more attention should go to differentiating between divergent conceptualizations of social capital in order to find the common ground and make comparison possible [29,58].

## Figures and Tables

**Table 1:** Descriptive statistics (*N* = 2730 respondents in *N* = 142 neighborhoods).

	Mean/Percentage	Standard Deviation	Min.	Max.
Self-rated health	4.06	0.79	1	5
**Individual characteristics**				
Gender				
Man	48.00%			
Woman	52.00%			
Age	47.97	18.76	18	95
Nationality				
Belgian	90.30%			
Other	9.70%			
Home ownership				
Owners	68.60%			
Tenants in social rented housing	10.50%			
Tenants in private rented housing	20.90%			
Education level				
Low	18.90%			
Middle	33.80%			
High	47.40%			
Individual social support	17.25	6.32	0	28
Social contacts per day				
≤10	44.62%			
11–25	25.27%			
≥26	30.11%			
**Neighborhood characteristics**				
Social trust	14.22	1.34	10.33	17.25
Informal social control	18.37	2.20	12.00	24.50
Disorder	12.62	3.26	6.38	20.63

**Table 2:** Bivariate correlations of neighborhood-level variables (*N* = 142).

	1	2	3
1. Social trust	1		
2. Informal social control	0.13 ^n.s.^	1	
3. Disorder	−0.57 ***	0.09 ^n.s.^	1

*** *p* < 0.001, n.s. = not significant.

**Table 3:** Estimates (and standard errors) of the hierarchical linear models explaining self-rated health (*N* = 2730).

	Model 0 B (S.E)	Model 1 B (S.E.)	Model 2 B (S.E.)	Model 3 B (S.E.)	Model 4 B (S.E.)
**Level 2: Neighborhood-level characteristics (fixed effects)**					
Social trust (standardized)			0.07 *** (0.02)	0.04 * (0.02)	0.04 * (0.02)
Informal social control (stand.)			n.s.	n.s.	n.s.
Disorder (standardized)				−0.06 ** (0.02)	−0.05 * (0.02)
**Level 1: Individual-level characteristics (fixed effects)**					
Gender (ref. cat. = women)		0.07 * (0.03)	0.07 * (0.03)	0.07 * (0.03)	0.06 * (0.03)
Age (standardized)		−0.21 *** (0.02)	−0.21 *** (0.02)	−0.21 *** (0.02)	−0.16 *** (0.02)
Nationality (ref. cat. = Belgian)		n.s.	n.s.	n.s.	n.s.
Education (ref. cat. = high)					
*Low*		−0.34 *** (0.04)	−0.34 *** (0.04)	−0.34 *** (0.04)	−0.27 *** (0.04)
*Middle*		−0.17 *** (0.03)	−0.17 *** (0.03)	−0.17 *** (0.03)	−0.12 *** (0.03)
Home ownership (ref. cat. = owner)					
*Tenants in social rented housing*		−0.25 *** (0.05)	−0.21 *** (0.05)	−0.18 *** (0.05)	−0.13 * (0.05)
*Tenants in private rented housing*		−0.10 ** (0.04)	−0.09 * (0.04)	−0.08 * (0.04)	n.s.
Individual social support (stand.)					0.11 *** (0.02)
Network size (ref. cat. = ≥26)					
*≤10*					-0.13 ** (0.04)
*11–25*					n.s.
Intercept	4.06 *** (0.02)	4.20 *** (0.03)	4.19 *** (0.03)	4.18 *** (0.03)	4.20 *** (0.04)
**Random effects**					
Neighborhood	0.018 **	0.006 n.s.	0.002 n.s.	0.001 n.s.	0.002 n.s.
Individual	0.610 ***	0.540 ***	0.539 ***	0.539 ***	0.524 ***
**ICC**	2.86%	1.18%	0.46%	0.24%	0.33%
**Δ Deviance (Δ df)**		425 (4) ***	8 (2) *	n.s. 3 (1)	163 (2) ***

*N* (level 1) = 2730, *N* (level 2) = 142; *** *p* < 0.001, ** *p* < 0.01, * *p* < 0.05, n.s. = not significant.

## Data Availability

The data presented in this study are available on request from the corresponding author. The data are not publicly available due to privacy reasons (cf. requirements of the Ethics Committee).

## Appendix A

**Table A1:** Results of factor and reliability analyses regarding scale construction for relevant indicators (Cronbach’s alpha’s in bold, factor loadings in italics).

Summative Scale and Individual Items	Coding	Cronbach’s Alpha/*Factor Loadings*
**Individual characteristics**		
*Individual social support* (individual items were on an 8-point scale)		**0.81**
How many friends, family members or acquaintances		
1. understand your problems?	0 → >10	*0.72*
2. would let you move into their house for a week if you temporarily could not stay at your house?	0 → >10	*0.72*
3. would encourage you to go to the doctor if you experience health problems?	0 → >10	*0.73*
4. make you feel good? (e.g., make you feel you are useful or make you feel they are glad to know you)	0 → >10	*0.70*
**Neighborhood characteristics**		
*Social trust* (individual items were on an 5-point Likert scale)		**0.80**
1. People around here are willing to help their neighbors	totally disagree → totally agree	*0.76*
2. This is a close-knit neighbourhood	totally disagree → totally agree	*0.76*
3. People in this neighborhood can be trusted	totally disagree → totally agree	*0.62*
4. Contacts between inhabitants in this neighborhood are generally positive	totally disagree → totally agree	*0.72*
*Informal social control* (individual items were on an 5-point Likert scale)		**0.84**
How likely is it that you could count on neighbors intervening when …		
1. children were skipping school and hang out on a street corner	very likely → very unlikely	*0.61*
2. children were spray-painting graffiti on a local building	very likely → very unlikely	*0.73*
3. children were showing disrespect to an adult	very likely → very unlikely	*0.69*
4. a fight breaks out in front of their house	very likely → very unlikely	*0.72*
5. children were making too much racket	very likely → very unlikely	*0.72*
6. children are using soft drugs (smoking weed, hasj, etc.)	very likely → very unlikely	*0.63*
*Disorder* (individual items were on an 5-point Likert scale)		**0.85**
1. Adolescents hanging around on street corners	never → very often	*0.66*
2. Groups of adolescents harassing people to obtain money or goods	never → very often	*0.78*
3. Men drinking alcohol in public	never → very often	*0.67*
4. People selling drugs (hash, weed, etc.) on the streets	never → very often	*0.70*
5. People being threatened on the streets with weapons or knives	never → very often	*0.65*
6. Fights between adolescents on the streets	never → very often	*0.80*

Cronbach’s alpha’s in bold, factor loadings in italics.

**Table A2:** Logistic regression coefficients (and standard errors) of a hierarchical ordered logit model (i.e., model for ordinal dependent variables, 2nd order PQL-estimation) explaining self-rated health, using ‘very good self-rated health’ as the reference category (*N* = 2730).

	Model Parallel to Model 4 in Table 3 B (S.E)
**Level 2: Neighborhood-level characteristics (fixed effects)**	
Social trust (standardized)	−0.102 * (0.049)
Informal social control (standardized)	n.s.
Disorder (standardized)	0.110 * (0.050)
**Level 1: Individual-level characteristics (fixed effects)**	
Gender (ref. cat. = women)	−0.178 * (0.077)
Age (standardized)	0.459 *** (0.046)
Nationality (ref. cat. = Belgian)	n.s.
Education (ref. cat. = high)	
*Low*	0.745 *** (0.114)
*Middle*	0.350 *** (0.088)
Home ownership (ref. cat. = owner)	
*Tenants in social rented housing*	0.339 * (0.137)
*Tenants in private rented housing*	n.s.
Individual social support (standardized)	−0.291 *** (0.043)
Network size (ref. cat. = ≥26)	
*≤10*	0.303 ** (0.103)
*11–25*	n.s.
Intercept (ref. cat. = very good self-rated health)	
*Very bad self-rated health*	−5.683 *** (0.246)
*Bad self-rated health or worse*	−3.884 *** (0.138)
*Moderate self-rated health or worse*	−2.107 *** (0.107)
*Good self-rated health or worse*	0.694 *** (0.096)
**Random effects**	
Neighborhood	0.005 n.s.

*N* (level 1) = 2730, *N* (level 2) = 142; *** *p* < 0.001, ** *p* < 0.01, * *p* < 0.05, n.s. = not significant. Estimated in MLwiN version 3.02.
